# Mental health status among women of reproductive age from underserved communities in the United States and the associations between depression and physical health. A cross-sectional study

**DOI:** 10.1371/journal.pone.0231243

**Published:** 2020-04-08

**Authors:** Sue C. Lin, Nadra Tyus, Maura Maloney, Bonnie Ohri, Alek Sripipatana

**Affiliations:** U.S. Department of Health and Human Services, Health Resources and Services Administration, Bureau of Primary Health Care, Office of Quality Improvement, Rockville, MD, United States of America; Columbia University, KAZAKHSTAN

## Abstract

**Background:**

In 2017, 46.6 million U.S. adults aged 18 or older self-reported as having mental illness of which 52.0% or 24.2 million are women age 18–49. Perinatal depression and anxiety are linked to adverse outcomes concerning pregnancy, maternal functioning, and healthy child development.

**Methods and findings:**

Using the 2014 Health Center Patient Survey (HCPS), the objectives of the cross-sectional study are to assess the prevalence of self-reported mental health conditions among female patients of reproductive age and to examine the association between depression and physical health. Physical health conditions of interest included self-rated health, obesity, hypertension, smoking, and diabetes, which all have established associations with potential pregnancy complications and fetal health. The study found 40.8% of patients reported depression; 28.8% reported generalized anxiety; and 15.2% met the criteria for serious psychological distress on the Kessler 6 scale. Furthermore, patients with depression had two to three times higher odds of experiencing co-occurring physical health conditions.

**Conclusions:**

This study expands the discourse on maternal mental health, throughout the preconception, post-partum, and inter-conception care periods to improve understanding of the inter-correlated physical and mental health issues that could impact pregnancy outcomes and life course trajectory. From 2014 to 2018, the Health Resources and Services Administration (HRSA) has supported investments of nearly $750 million to improve and expand access to mental health and substance use disorder services for prevention, treatment, health education and awareness through comprehensive primary care integration. Moving forward, HRSA will implement strategic training and technical assistance (T/TA) framework that is designed to accelerate the adoption of science driven solutions in primary care in addressing depression for patients with co-occurring chronic conditions and advancing positive maternal outcomes.

## Introduction

In 2017, an estimated 46.6 million U.S. adults aged 18 or older and 24.2 million or 52.0% are women age 18–49 self-reported as having mental illness [1]. Perinatal depression and anxiety are widely prevalent and linked to adverse outcomes concerning pregnancy, maternal functioning, and healthy child development [2–4]. In addition, depressive disorder is a common comorbid condition associated with chronic diseases such as: asthma, arthritis, cardiovascular disease, diabetes, obesity, chronic obstructive pulmonary disorder, cancer, and autoimmune disorders [5–10]. In particular, poor mental health among women has been associated with future chronic diseases such as diabetes and heart disease [11].

The directional relationship between physical health and depression in adults is a complex health issue, making it difficult to determine a causal link [6, 7]. Some evidence suggests that depressive disorders in patients can precipitate chronic disease due to an increased prevalence of harmful health behaviors such as smoking, excess alcohol consumption, inadequate nutrition, and lack of physical activity, all of which increase the risk of chronic diseases [6, 12, 13]. On the other hand, chronic diseases can also exacerbate the health among women of reproductive age who have depression, resulting in deteriorating mental health status while increasing the risk of postpartum depression. In particular, women with asthma, obesity, and non-gestational diabetes are at a higher risk for postpartum depression, compared to women who show no symptoms of these diseases [14–16]. Furthermore, the substantial excess mortality for both natural and unnatural causes of death among adults with mental illness, in which patients with anxiety or depressive disorders died 7.9 years earlier than the general population, is a public health issue of significant concern [17–19].

A clearer understanding of physical health factors associated with mental health conditions in women of reproductive age is critical to improving clinical strategies and access to mental health screening and treatment in the primary care setting. Among non-pregnant women aged 18 to 44 nationally, 3.1% had diabetes, 10.9% had hypertension, 55.1% were overweight or obese, and 16.9% were current smokers [20]. Given the high prevalence, increased awareness of how chronic disease risk factors relate to mental health can improve timely diagnosis and treatment of depressive disorders the impact of chronic disease.

Both domestically and globally, women reported significantly poorer health than men in health related quality of life, depression, and psychological distress [21–24]. As socioeconomic inequalities and sociodemographic variations contribute to poorer health status, it is important to understand the association between self-reported health, which is a common measure of general health, by women of reproductive age and their mental health especially among vulnerable populations [25–27].

In the United States, many women from underserved communities located in geographic areas that lack of access to primary care services receive care from health centers (HC) funded by the Health Resources and Services Administration (HRSA). By federal statute, HCs must provide primary health care services including prenatal and perinatal services [28]. The interdisciplinary clinical and administrative staff at HC deliver culturally competent and comprehensive primary care as well as enabling services such as health education, translation, and transportation that support access to health care. In fact, nearly 30% of the over 27 million HC patients are women of reproductive ages (15 to 49 years), which warrants the enhanced emphasis on the health and well-being of mothers and would-be mothers served at HCs [29]. However, there are challenges to ensuring optimal health trajectories for these patients. By design, the Health Center Program (HCP) mission is to provide care to the nation’s underserved and vulnerable populations where the majority of HC patients are poor, uninsured, or underinsured; specifically, nine out of ten patients are at 200% or below the federal poverty level (FPL), and over 70% are uninsured or covered by Medicaid [29]. FPL is a measure of income issued every year by the United States Department of Health and Human Services (HHS) to determine eligibility for certain public programs, health insurance, and benefits [30]. This study will assess the prevalence of mental health conditions including depression, generalized anxiety, panic disorder, schizophrenia, bipolar disorder, and serious psychological distress among female patients of reproductive age; furthermore, the study will examine the association between depression and physical health including self-rated health, obesity, hypertension, smoking, and diabetes that will inform and improve the future delivery of primary care and behavioral health care services in HCs.

## Methods

### IRB approval

Institutional Review Board (IRB) approval was obtained by Research Triangle International (RTI), which administered the 2014 Health Center Patient Survey (HCPS). The HCPS is a de-identified survey dataset that comes from in-person, one-on-one interviews with self-report by patients, and are nationally representative of the Health Center Program patient population. No patient records are included in the HCPS dataset. Once patients were sampled and recruited into the HCPS, the informed consent process was administered to obtain the required permission to conduct interviews with respondents from each age group and the type of interview conducted. Interviews were not conducted unless the permission and consent requirements had been met. The informed consent form was read aloud to each participant. Spanish, Chinese, Vietnamese, and Korean versions of the consent forms were available for use by certified bilingual field interviewers only for sample members who preferred to conduct the interview in these languages.

### Data source

We conducted statistical analyses using data from the 2014 HCPS, which provided information on a nationally representative sample of HC patients. The HCPS surveyed 7,002 HC patients from October 2014 to April 2015 with a final response rate of 91.4% [31]. HCPS used a stratified three-stage random sampling design for sample selection. The first-stage sampling units were comprised of HRSA funded HC organizations across the United States. The second-stage sampling units were eligible primary care delivery sites within the HC organization. Finally, the third-stage sampling units were eligible patients who had at least one medical visit in the past 12 months to an eligible HC site. Trained interviewers conducted computer assisted personal interviews (CAPI) in several languages including English, Spanish, Mandarin, Cantonese, Korean, and Vietnamese. HCPS survey questions were modeled from related national surveys including National Health Interview Survey (NHIS), National Ambulatory Medical Care Survey (NAMCS), Medical Expenditure Panel Survey (MEPS), and National Health and the Nutrition Examination Survey (NHANES) and focused on sociodemographic characteristics, health conditions, health behaviors, access to, and utilization of, health care services, and satisfaction with health care.

### Sample

The World Health Organization defines women of reproductive age as females aged 15 to 49 years. We excluded male patients and female patients who were not of reproductive age from the total group of HCPS 7,002 respondents. The final analytic sample was N = 2,061.

### Measures

We explored the sociodemographic characteristics of HC patients, including race/ethnicity (non-Hispanic White [NHW], Hispanic/Latino, non-Hispanic Black [NHB], and non-Hispanic “other”), geography (urban, rural), household poverty level (≤100% FPL, 101–199% FPL, and ≥200 FPL), marital status, age group by 5 year intervals (15–19, 20–24, 25–29, 30–34, 35–39, 40–44, and 45–49), education level (less than high school, high school, and more than high school) and insurance status (Medicaid, Other Third Party, and Uninsured). Medicaid is a public health insurance program for individuals with low income or disabilities. Other third party insurance may include employer sponsored health insurance, private insurance purchased directly by individuals, and health care insurance program for military uniformed service members, retirees, and their families.

Mental health conditions were assessed through the HCPS mental health questionnaire module, in which respondents were asked whether they have the following conditions: “Has a doctor or other health professional ever told you that you had depression, generalized anxiety, panic disorder, schizophrenia, or bipolar disorder?” In addition, the module included the Kessler 6 (K6) nonspecific distress scale, which is a 6-item, psychological screening instrument to identify individuals with serious psychological distress (SPD) [32]. The K6 responses ranged from “none of the time” coded 0 to “all of the time” coded 4, which yielded a total score between 0 and 24 with the cut-point ≥ 13 for SPD.

In addition, we examined physical health through self-rated health (SRH) and conditions that included obesity, hypertension, smoking, and diabetes. Obesity was assessed by body weight index calculated from responses to questions on height and weight: 1) How tall are you without shoes; and 2) How much do you weigh without clothes or shoes? Respondents answered the following questions for other physical health conditions: 1) Have you ever been told by a doctor or other health professional that you had hypertension, also called high blood pressure?; 2) Do you now smoke cigarettes every day, some days or not at all?; and, 3) Have you ever been told by a doctor or health professional that you had diabetes or sugar diabetes. Self-rated health is measure of general health with the response items of “excellent,” “very good,” “good,” “fair,” or “poor.” The conditions included in the analysis have been associated with potential pregnancy complications and fetal health [33–36].

### Data analysis

We conducted descriptive analyses to determine the distribution of sociodemographic characteristics among female HC patients of reproductive age. We further examined the prevalence of mental health conditions reported by female patients of reproductive age for depression, generalized anxiety, panic disorder, schizophrenia, bipolar disorder, and SPD. Analyses were weighted based upon the complex sampling design, which included stratification, clustering, and multistage sampling, to produce weighted frequencies that are representative of the HC patient population [37]. We then conducted bivariate analyses to determine the empirical relationship between depression and physical health by SRH, obesity, hypertension, smoking, and diabetes as well as substance use disorder (SUD). High risk for Substance Use Disorder (SUD) in alcohol, drug dependence, and non-medical use of opioids was assessed using the Alcohol, Smoking and Substance Involvement Screening Test (ASSIST), which was developed through the World Health Organization (WHO) to identify and treat SUD in primary care settings. In addition, we looked at the sociodemographic characteristics distributions of female patients of reproductive age with and without depression. Finally, multivariate logistic regression models examined the association between depression and SRH as well as physical health conditions of obesity, hypertension, smoking, and diabetes. The model controlled for race/ethnicity, geography, household income, marital status, SUD, and health insurance. All analyses were conducted using SAS version 9.3.

## Results

**Table 1** described the sociodemographic characteristics of all female patients of reproductive age between the ages of 15 to 49 years served at HCs. The data showed the following: 41.7% reported to be NHW; 51.6% lived in urban geographical areas; 60.2% had household incomes at or below the 100% FPL; 25.0% are married; 34.8% had education beyond high school; and finally, 31.9% or 2.3 million patients were uninsured.

**Table 1:** Sociodemographic characteristics of female patients of reproductive age (15–49 yrs) served at HRSA funded health centers.

Characteristics (N = 2061)	Frequency	Weighted Frequency	Weighted Percentage	SE
**Race/ethnicity**				
Hispanic	879	2,254,171	31.3%	2.16
Non-Hispanic White	414	3,007,926	41.7%	2.49
Non-Hispanic Black	440	1,517,601	21.0%	1.86
Other	328	433,460	6.0%	0.93
**Geography**				
Urban	1374	3,723,822	51.6%	2.44
Rural	687	3,489,336	48.4%	2.44
**Household federal poverty level (FPL)**				
≤100% FPL	1296	4,046,888	60.2%	2.46
101–199% FPL	516	1,859,448	27.7%	2.21
≥200% FPL	169	817,467	12.2%	1.78
**Married**	590	1,804,364	25.0%	2.03
**Age Group (years)**				
15–19	144	970,007	13.4%	1.88
20–24	222	1,262,629	17.5%	1.97
25–29	336	1,030,929	14.3%	1.54
30–34	350	1,351,404	18.7%	1.96
35–39	323	1,152,248	16.0%	1.84
40–44	318	766,319	10.6%	1.27
45–49	368	679,622	9.4%	1.15
**Education**				
Less than high school	908	2,674,837	37.1%	2.37
High school	534	2,015,806	28.0%	2.20
More than high school	614	2,508,631	34.8%	2.29
**Insurance status**				
Medicaid	966	3,674,703	50.9%	2.44
Other Third Party	482	1,234,437	17.1%	1.63
Uninsured	613	2,304,018	31.9%	2.34

Source: 2014 Health Center Patient Survey

**Table 2** delineated the mental health conditions reported by female HC patients of reproductive age. The data demonstrated the following: 40.8% or 2.9 million patients reported depression; 28.8% patients reported generalized anxiety, which represented just over 2 million patients; and 15.2% of patients or over a million met the criteria for SPD as determined by the K6 screening scale.

**Table 2:** Mental health conditions of female patients of reproductive age (15–49 yrs) served at HRSA funded health centers.

Mental Health Status	Frequency	Weighted Frequency	Weighted Percentage	SE
Depression	807	2,942,872	40.8%	2.41
Generalized anxiety	573	2,077,316	28.8%	2.26
Panic disorder	294	1,122,807	15.6%	1.82
Schizophrenia	68	140,245	1.9%	0.55
Bipolar disorder	260	919,815	12.8%	1.65
Serious Psychological Distress	306	1,092,852	15.2%	1.83

Source: 2014 Health Center Patient Survey

Since depression was found to be the most common mental health condition among female HC patients of reproductive age, **Table 3** further examined the bivariate analysis of patients with depression and sociodemographic characteristics. 64.6% of NHW reported depression. For patients living in rural areas, 57.7% were depressed. With respect to insurance types, 57.7% of Medicaid patients reported depression in comparison to 16.6% among patients with other third party insurance. 22.5% of patients with depression were married.

**Table 3:** Sociodemographic characteristics of female patients with depression of reproductive age (15–49 yrs) served at HRSA funded health centers.

	Patients with Depression		Patients without Depression		Chi-Square P-Value
Characteristics (N = 2061)	Weighted %	SE	Weighted %	SE	
**Race/ethnicity**					<0.01
Hispanic	17.0%	2.42	41.1%	3.04	
Non-Hispanic White	64.6%	3.40	25.9%	2.99	
Non-Hispanic Black	14.7%	2.43	25.4%	2.62	
Other	3.6%	0.98	7.7%	1.40	
**Geography**					<0.01
Urban	42.3%	3.73	58.1%	3.11	
Rural	57.7%	3.73	41.9%	3.11	
**Household federal poverty level (FPL)**					0.34
≤100% FPL	61.9%	3.91	58.9%	3.16	
101–199% FPL	28.9%	3.61	26.7%	2.75	
≥200% FPL	9.2%	2.58	14.3%	2.43	
**Insurance status**					0.04
Medicaid	57.7%	3.83	46.3%	3.12	
Other Third Party	16.6%	2.66	17.4%	2.07	
Uninsured	25.7%	3.50	36.2%	3.09	
**Married**	22.5%	3.12	26.8%	2.67	0.31

Source: 2014 Health Center Patient Survey

**Table 4** assessed the bivariate frequency distribution of patients with, and without, depression with SRH and physical conditions. The data showed that 47.8% of patients with depression and 27.0% of patients without depression reported fair or poor SRH (p-value <0.01). 61.0% of patients with depression were obese as compared to 39.2% of patients that were not depressed (p-value<0.01). In addition, 30.3% of patients with depression had hypertension as opposed to 13.5% of patients without depression (p-value <0.01); 42.9% of patients with depression were current smokers in comparison to 13.2% (p-value <0.01); and finally, 16.9% and 6.3% of patients with and without depression had diabetes, respectively (p-value <0.01). 2.0% of patients with depression had SUD (p-value = 0.44).

**Table 4:** Physical health status and conditions of female patients with depression and of reproductive age (15–49 yrs) served at HRSA funded health centers.

	Patients with Depression		Patients without Depression		Chi-Square P-Value
	Weighted %	SE	Weighted %	SE	
**Health Status**					< .01
Excellent	4.0%	1.28	9.0%	1.65	
Very Good	5.7%	1.83	15.6%	2.23	
Good	42.5%	3.89	48.5%	3.13	
Fair/Poor	47.8%	3.88	27.0%	2.67	
**Body Mass Index**					< .01
Obese	61.0%	3.79	39.2%	3.07	
Overweight	19.9%	3.01	26.4%	2.78	
Neither Overweight or Obese	19.0%	3.01	34.3%	3.12	
**Hypertension**	30.3%	3.65	13.5%	2.18	< .01
**Smoking**	42.9%	3.95	13.2%	2.28	< .01
**Diabetes**	16.9%	3.22	6.3%	1.26	< .01
**Substance Use Disorder**	2.0%	0.73	1.1%	0.70	0.44

Source: 2014 Health Center Patient Survey

**Table 5** contained the multivariate logistic regression of the association between depression and physical health status. Female patients of reproductive age with depression had more than three times the odds of reporting smoking (adjusted odds ratio [aOR] = 3.02, 95% confidence interval [CI], 1.79 to 5.10) and diabetes (aOR = 3.11, 95% C, 1.70 to 5.70). Furthermore, they had more than two times the odds of having fair or poor SRH (aOR = 2.95, 95% CI, 1.84 to 4.71), obesity (aOR = 2.59, 95% CI, 1.64 to 4.08), and hypertension (aOR = 2.48, 95% CI, 1.40 to 4.39).

**Table 5:** Multivariate logistic regression of associations between depression and physical health status and conditions among female patients with depression of reproductive age (15–49 yrs) served at HRSA funded health centers.

	aOR	95% CI
**Fair/Poor Health Status**	2.95	(1.84–4.71)
**Obesity**	2.59	(1.64–4.08)
**Hypertension**	2.48	(1.40–4.39)
**Smoking**	3.02	(1.79–5.10)
**Diabetes**	3.11	(1.70–5.70)

Source: 2014 Health Center Patient Survey Model controlled for race/ethnicity, geography, household income, insurance, marital status, and substance use disorder

## Discussion

This study expands the discourse on maternal health, particularly around mental health, throughout the preconception, post-partum, and inter-conception care periods. Study findings advance understanding of inter-correlated health issues of female HC patients of reproductive age that could impact their health during pregnancy and their life course trajectory. According to the 2017 NHIS, the prevalence of SPD in women ages 18 to 44 was 3.6 percent in the general population [37]. Among female HC patients of reproductive age, the prevalence of SPD is over 300% greater than the general population, which further underscores the importance of supporting primary care behavioral health integration in HC to address patients’ behavioral health needs. Studies have demonstrated that the presence of SPD is associated with increased mortality, activity limitation, and decreased health-related quality of life [38–40]. The disproportionate prevalence and mental health burden of SPD among HC patients illustrates the importance of primary care behavioral health integration and chronic care management in order to effectively address mental health and comorbid chronic diseases [41–46].

The study found female patients with self-reported depression exhibited two to three times higher odds of experiencing co-occurring physical health conditions compared to their counterparts without self-reported depression, after accounting for potential confounders. Some of these physical health conditions could become more complicated with pregnancy, given the physiological and metabolic changes associated with pregnancy. To that end, the results suggest the importance of addressing health issues, including enhanced chronic disease management, smoking cessation counseling, and healthy weight counseling, for female patients of childbearing age.

**Fig 1:**
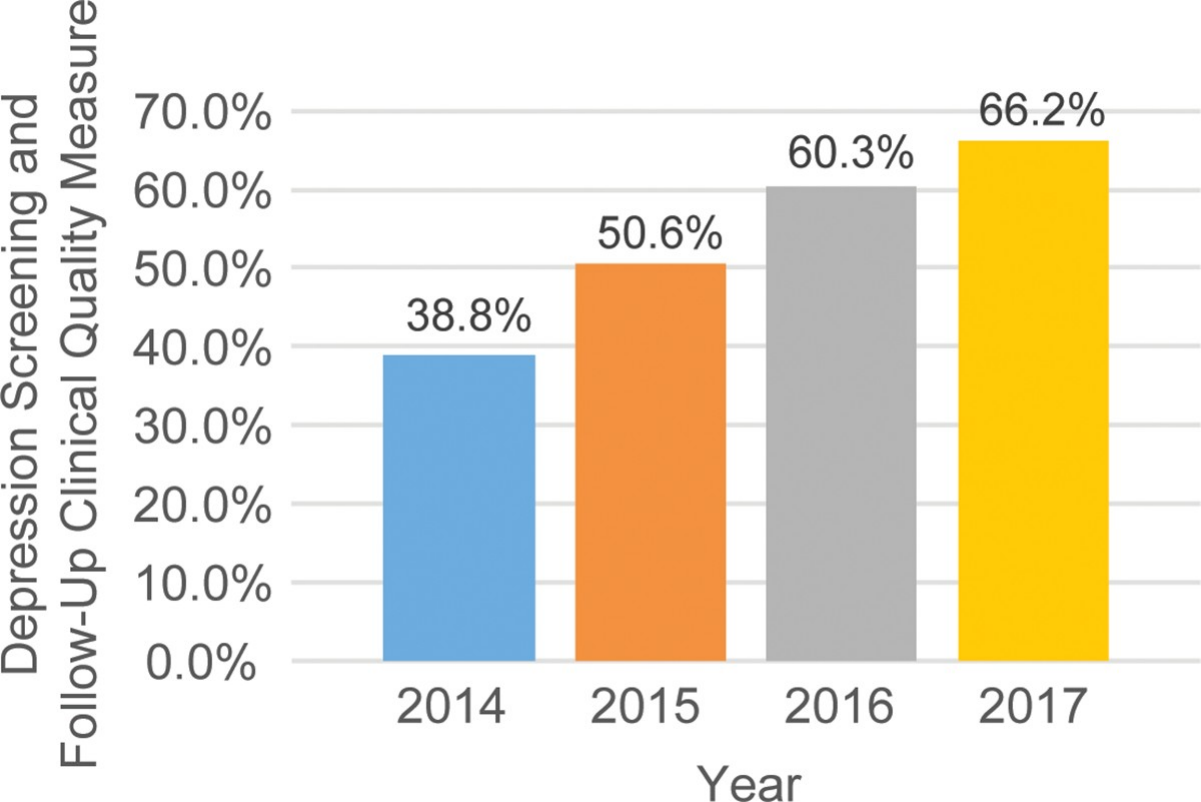
Trend in depression screening and follow-up clinical quality measure performance.

Given the focus of HCs on prevention, chronic disease management, and care coordination, HC providers and care teams can be further trained and supported to accelerate the implementation of innovative promising practices and evidence-based interventions in women’s health. In fact, the health conditions found to be correlated with depression are among a set of conditions that are a priority for the HCP, and are included among the core set of 16 clinical quality measures (CQM) that HCs are required to report annually into the Uniform Data System (UDS). The depression screening and follow-up measure was added to UDS in 2014 to monitor the quality of mental health services available in HCs. Through continuous quality improvement efforts at the national, state, and clinic level, the national average for the depression CQM has increased from 38.8% in 2014 to 66.2% in 2017 in HCs as shown by Fig 1. Optimization of clinical workflow and health IT infrastructure also holds the potential to enhance HC primary care teams’ capacity in providing timely and high quality care. Effective care management through incorporation of patient health education, patient engagement in treatment and social services supports, as well as appropriate evidence-based pharmacotherapy or psychotherapy in the treatment of patients with chronic diseases and depression have been associated with increased use of antidepressants, reduced utilization of emergency services, and improved patient outcomes [38–41].

Our study has several limitations to consider. First, the HCPS is a cross-sectional dataset and, therefore, we cannot make causal inferences regarding whether these mental health conditions existed prior to onset of the physical health conditions. Second, there is a potential for recall bias in patient self-report of mental health conditions, SRH and physical health conditions. Third, the HCPS patients were queried in-person by a survey interviewer, which could potentially introduce social desirability bias on responses, which would be perceived more positively by the interviewer.

### Call to action: Accelerate primary care behavioral HEALTH INTEGRATION through strategic training and technical assistance support

The research findings of high prevalence of depression and serious psychological distress as well as the strong associations between depression and chronic diseases among female HC patients of reproductive age warrants the enhanced emphasis on women’s health as well as the innovative care and treatment of co-occurring physical and mental health conditions among HCs patients. Furthermore, the findings have implications for clinical practice, technical assistance for HC, primary care integration models, and prevention activities to reduce risks to women of reproductive age who are at risk for negative maternal and birth outcomes. Recognizing the time constraints of primary care teams in provision of preventive services, management of chronic disease, adherence to the latest clinical guidelines, and the need for knowledge translation through implementation science in improving the delivery patient-centered care, we propose a training and technical assistance (T/TA) framework moving forward. This T/TA framework is designed to accelerate science driven solutions as well as health center innovations for primary care behavioral health integration in the following four areas: 1) Facilitating rapid adoption of innovative primary care integrated models for behavioral health; 2) Supporting health center workforce training; 3) Enhancing clinical workflow and health informatics; and 4) Improving primary care service to system coordination and financial sustainability. The T/TA framework will support HCs and primary care team in service expansion, enhanced implementation and transformation of primary care behavioral health integration services, as well as augmented provider training and team-based care capacity for delivery of prevention, early diagnosis, and treatment.

From 2014 to 2018, HRSA has supported investments of nearly $750 million to improve and expand the delivery and access of mental health services and substance use disorder services for prevention, treatment, health education and awareness through comprehensive primary care integration. In conjunction with the service expansion supplements, HRSA has supported several training and technical assistance initiatives including the Center for Integrated Health Solutions (CIHS) jointly funded by the Substance Abuse and Mental Health Services Administration (SAMHSA) and HRSA, Opioid Addiction Treatment Project ECHO (Extension for Community Healthcare Outcomes) and the HRSA Center of Excellence in Behavioral Health Technical Assistance to advance the development and enhancement of integrated primary and behavioral health services to better address the needs of health center patients with mental health and substance use conditions, in particular opioid use disorder. To date, HCs have improved their capacity to provide mental health services, including a 67% increase in mental health workforce and a 59% increase in mental health patient visits [29]. The expansion of behavioral health services in HCs has provided an unprecedented investment in the primary care safety net to address this public health priority that will built upon ongoing investments in evidence-based intervention of patient centered medical home transformation, patient health education, patient navigation, medication therapy management, psychosocial peer supports, and augmentation of enabling services; however, much work remains [42–46]. With the increasing importance of the interdisciplinary primary care team in addressing behavioral health needs, strategic T/TA will enhance HC organizational and workforce capacity for treatment, efficient clinical workflow, data-informed clinical quality improvement, and care coordination with social service systems to support all patients with co-occurring chronic and mental health conditions including the advancement of positive maternal outcomes.

## References

[1] Results from the 2017 National Survey on Drug Use and Health:Detailed Tables Rockville, MD: Center for Behavioral Health Statistics and Quality, Substance Abuse and Mental Health Services Administration [cited 2020 February 14]. Available from: https://www.samhsa.gov/data/sites/default/files/cbhsq-reports/NSDUHDetailedTabs2017/NSDUHDetailedTabs2017.htm#tab8-33A.

[2] Ko JY, Farr SL, Dietz PM, Robbins CL. Depression and treatment among U.S. pregnant and nonpregnant women of reproductive age, 2005–2009. Journal of women's health (2002). 2012;21(8):830–6. 10.1089/jwh.2011.3466 PMC4416220 22691031

[3] O'Hara MW, Wisner KL. Perinatal mental illness: definition, description and aetiology. Best practice & research Clinical obstetrics & gynaecology. 2014;28(1):3–12. 24140480 10.1016/j.bpobgyn.2013.09.002 PMC7077785

[4] Ko JY, Rockhill KM, Tong VT, Morrow B, Farr SL. Trends in Postpartum Depressive Symptoms—27 States, 2004, 2008, and 2012. MMWR Morbidity and mortality weekly report. 2017;66(6):153–8. 10.15585/mmwr.mm6606a1 28207685 PMC5657855

[5] Carey M, Small H, Yoong SL, Boyes A, Bisquera A, Sanson-Fisher R. Prevalence of comorbid depression and obesity in general practice: a cross-sectional survey. The British journal of general practice: the journal of the Royal College of General Practitioners. 2014;64(620):e122–7. 24567650 10.3399/bjgp14X677482 PMC3933857

[6] Chapman DP PG, Strine TW. The Vital Link Between Chronic Disease and Depressive Disorders. Prev Chronic Dis. 2005;2(1). PMC1323317 15670467

[7] Euesden J, Danese A, Lewis CM, Maughan B. A bidirectional relationship between depression and the autoimmune disorders–New perspectives from the National Child Development Study. PLoS ONE. 2017;12(3):e0173015 10.1371/journal.pone.0173015 28264010 PMC5338810

[8] Halaris A. Comorbidity between depression and cardiovascular disease. International angiology: a journal of the International Union of Angiology. 2009;28(2):92–9. 19367238

[9] Simon GE. Treating depression in patients with chronic disease. Western Journal of Medicine. 2001;175(5):292–3. 10.1136/ewjm.175.5.292 11694462 PMC1071593

[10] Doyle T, Palmer S, Johnson J, Babyak MA, Smith P, Mabe S, et al Association of anxiety and depression with pulmonary-specific symptoms in chronic obstructive pulmonary disease. International journal of psychiatry in medicine. 2013;45(2):189–202. 10.2190/PM.45.2.g 23977821 PMC4005783

[11] Farr SL, Hayes DK, Bitsko RH, Bansil P, Dietz PM. Depression, diabetes, and chronic disease risk factors among US women of reproductive age. Preventing chronic disease. 2011;8(6):A119 22005612 PMC3221561

[12] Bartlem KM, Bowman JA, Freund M, Wye PM, McElwaine KM, Wolfenden L, et al Care Provision to Prevent Chronic Disease by Community Mental Health Clinicians. American Journal of Preventive Medicine. 2014;47(6):762–70. 10.1016/j.amepre.2014.08.003 25455118

[13] Stanley S, Laugharne J. The impact of lifestyle factors on the physical health of people with a mental illness: a brief review. International journal of behavioral medicine. 2014;21(2):275–81. 10.1007/s12529-013-9298-x 23443909

[14] Blais L, Salah Ahmed SI, Beauchesne M-F, Forget A, Kettani F-Z, Lavoie K. Risk of postpartum depression among women with asthma. The Journal of Allergy and Clinical Immunology: In Practice. 2018. 10.1016/j.jaip.2018.09.026 30292921

[15] Miller ES, Peri MR, Gossett DR. The association between diabetes and postpartum depression. Archives of women's mental health. 2016;19(1):183–6. 10.1007/s00737-015-0544-x 26184833

[16] Molyneaux E, Poston L, Ashurst-Williams S, Howard LM. Obesity and mental disorders during pregnancy and postpartum: a systematic review and meta-analysis. Obstetrics and gynecology. 2014;123(4):857–67. 10.1097/AOG.0000000000000170 24785615 PMC4254698

[17] Lawrence D, Kisely S, Pais J. The Epidemiology of Excess Mortality in People with Mental Illness. The Canadian Journal of Psychiatry. 2010;55(12):752–60. 10.1177/070674371005501202 21172095

[18] Pratt LA, Druss BG, Manderscheid RW, Walker ER. Excess mortality due to depression and anxiety in the United States: results from a nationally representative survey. General hospital psychiatry. 2016;39:39–45. 10.1016/j.genhosppsych.2015.12.003 26791259 PMC5113020

[19] Miller C, Bauer MS. Excess mortality in bipolar disorders. Current psychiatry reports. 2014;16(11):499 10.1007/s11920-014-0499-z 25194314

[20] Robbins C, Boulet SL, Morgan I, D'Angelo DV, Zapata LB, Morrow B, et al Disparities in Preconception Health Indicators—Behavioral Risk Factor Surveillance System, 2013–2015, and Pregnancy Risk Assessment Monitoring System, 2013–2014. Morbidity and mortality weekly report Surveillance summaries (Washington, DC: 2002). 2018;67(1):1–16. 10.15585/mmwr.ss6701a1 PMC5829866 29346340

[21] Boerma T, Hosseinpoor AR, Verdes E, Chatterji S. A global assessment of the gender gap in self-reported health with survey data from 59 countries. BMC Public Health. 2016;16:675 10.1186/s12889-016-3352-y 27475755 PMC4967305

[22] Williams G, Di Nardo F, Verma A. The relationship between self-reported health status and signs of psychological distress within European urban contexts. European journal of public health. 2017;27(suppl_2):68–73. 10.1093/eurpub/ckx008 28449045

[23] Cherepanov D, Palta M, Fryback DG, Robert SA. Gender differences in health-related quality-of-life are partly explained by sociodemographic and socioeconomic variation between adult men and women in the US: evidence from four US nationally representative data sets. Quality of life research: an international journal of quality of life aspects of treatment, care and rehabilitation. 2010;19(8):1115–24. 10.1007/s11136-010-9673-x PMC2940034 20496168

[24] Barry LC, Allore HG, Guo Z, Bruce ML, Gill TM. Higher burden of depression among older women: the effect of onset, persistence, and mortality over time. Archives of general psychiatry. 2008;65(2):172–8. 10.1001/archgenpsychiatry.2007.17 18250255 PMC2793076

[25] Cherepanov D, Palta M, Fryback DG, Robert SA, Hays RD, Kaplan RM. Gender differences in multiple underlying dimensions of health-related quality of life are associated with sociodemographic and socioeconomic status. Medical care. 2011;49(11):1021–30. 10.1097/MLR.0b013e31822ebed9 21945974 PMC3687080

[26] Kessler RC, Bromet EJ. The epidemiology of depression across cultures. Annual review of public health. 2013;34:119–38. 10.1146/annurev-publhealth-031912-114409 23514317 PMC4100461

[27] Bombak AE. Self-rated health and public health: a critical perspective. Frontiers in public health. 2013;1:15 10.3389/fpubh.2013.00015 24350184 PMC3855002

[28] Health Center Program Statute: Section 330 of the Public Health Service Act (42 U.S.C. §254b) [cited 2018 November 23]. Available from: http://uscode.house.gov/view.xhtml?req=granuleid:USC-prelim-title42-section254b&num=0&edition=prelim.

[29] Health Resources and Service Administration. 2018 Uniform Data System [Available from: https://bphc.hrsa.gov/uds/datacenter.aspx.

[30] Annual Update of the HHS Poverty Guidelines -A Notice by the Health and Human Services Department on 01/17/2020 [cited 2020 February 15]. Available from: https://www.federalregister.gov/documents/2020/01/17/2020-00858/annual-update-of-the-hhs-poverty-guidelines.

[31] Health Resources and Services Administration. 2014 Health Center Patient Survey Data File User’s Manual. 2016 [cited 2018 October 7]. Available from: https://bphc.hrsa.gov/datareporting/research/hcpsurvey/2014usermanual.pdf.

[32] Kessler RC, Barker PR, Colpe LJ, Epstein JF, Gfroerer JC, Hiripi E, et al Screening for serious mental illness in the general population. Archives of general psychiatry. 2003;60(2):184–9. 10.1001/archpsyc.60.2.184 12578436

[33] Patro Golab B, Santos S, Voerman E, Lawlor DA, Jaddoe VWV, Gaillard R. Influence of maternal obesity on the association between common pregnancy complications and risk of childhood obesity: an individual participant data meta-analysis. The Lancet Child & adolescent health. 2018. 10.1016/S2352-4642(18)30273-6 PMC6196075 30201470

[34] Mund M, Louwen F, Klingelhoefer D, Gerber A. Smoking and pregnancy—a review on the first major environmental risk factor of the unborn. International journal of environmental research and public health. 2013;10(12):6485–99. 10.3390/ijerph10126485 24351784 PMC3881126

[35] Brown HM, Green ES, Tan TCY, Gonzalez MB, Rumbold AR, Hull ML, et al Periconception onset diabetes is associated with embryopathy and fetal growth retardation, reproductive tract hyperglycosylation and impaired immune adaptation to pregnancy. Scientific reports. 2018;8(1):2114 10.1038/s41598-018-19263-8 29391475 PMC5794861

[36] Wenger NK, Arnold A, Bairey Merz CN, Cooper-DeHoff RM, Ferdinand KC, Fleg JL, et al Hypertension Across a Woman's Life Cycle. Journal of the American College of Cardiology. 2018;71(16):1797–813. 10.1016/j.jacc.2018.02.033 29673470 PMC6005390

[37] 2014 Health Center Patient Survey Data File User’s Manual [cited 2020 February 16]. Available from: https://bphc.hrsa.gov/datareporting/research/hcpsurvey/2014usermanual.pdf.

[38] de Lusignan S, Chan T, Tejerina Arreal MC, Parry G, Dent-Brown K, Kendrick T. Referral for psychological therapy of people with long term conditions improves adherence to antidepressants and reduces emergency department attendance: controlled before and after study. Behaviour research and therapy. 2013;51(7):377–85. 10.1016/j.brat.2013.03.004 23639304 PMC3677087

[39] Dube SR, Caraballo RS, Dhingra SS, Pearson WS, McClave AK, Strine TW, et al The relationship between smoking status and serious psychological distress: findings from the 2007 Behavioral Risk Factor Surveillance System. International Journal of Public Health. 2009;54(1):68–74. 19396580 10.1007/s00038-009-0009-y

[40] Unutzer J, Park M. Strategies to improve the management of depression in primary care. Primary care. 2012;39(2):415–31. 10.1016/j.pop.2012.03.010 22608874 PMC4127627

[41] Thomas M, Hutchison M, Castro G, Nau M, Shumway M, Stotland N, et al Meeting Women Where They Are: Integration of Care As the Foundation of Treatment for At-Risk Pregnant and Postpartum Women. Maternal and child health journal. 2017;21(3):452–7. 10.1007/s10995-016-2240-5 28168590

[42] Rodis JL, Sevin A, Awad MH, Porter B, Glasgow K, Hornbeck Fox C, et al Improving Chronic Disease Outcomes Through Medication Therapy Management in Federally Qualified Health Centers. J Prim Care Community Health. 2017;8(4):324–31. 10.1177/2150131917701797 28381095 PMC5932724

[43] Roland KB, Milliken EL, Rohan EA, DeGroff A, White S, Melillo S, et al Use of Community Health Workers and Patient Navigators to Improve Cancer Outcomes Among Patients Served by Federally Qualified Health Centers: A Systematic Literature Review. Health Equity. 2017;1(1):61–76. 10.1089/heq.2017.0001 28905047 PMC5586005

[44] Komaromy M, Duhigg D, Metcalf A, Carlson C, Kalishman S, Hayes L, et al Project ECHO (Extension for Community Healthcare Outcomes): A new model for educating primary care providers about treatment of substance use disorders. Subst Abus. 2016;37(1):20–4. 10.1080/08897077.2015.1129388 26848803 PMC4873719

[45] Dobbins JM, Peiper N, Jones E, Clayton R, Peterson LE, Phillips RL Jr. Patient-Centered Medical Home Recognition and Diabetes Control Among Health Centers: Exploring the Role of Enabling Services. Popul Health Manag. 2018;21(1):6–12. 10.1089/pop.2017.0001 28467266

[46] Shi L, Lee DC, Chung M, Liang H, Lock D, Sripipatana A. Patient-Centered Medical Home Recognition and Clinical Performance in U.S. Community Health Centers. Health Serv Res. 2017;52(3):984–1004. 10.1111/1475-6773.12523 27324440 PMC5441497

